# Metabolic profiling of the synthetic cannabinoid APP-CHMINACA (PX-3) as studied by in vitro and in vivo models

**DOI:** 10.1007/s11419-024-00705-0

**Published:** 2024-11-22

**Authors:** Cristian Camuto, Fabio De- Giorgio, Giorgia Corli, Sabrine Bilel, Monica Mazzarino, Matteo Marti, Francesco Botrè

**Affiliations:** 1 Laboratorio Antidoping FMSI, Largo Giulio Onesti 1, 00197 Rome, Italy; 2 Department of Health Care Surveillance and Bioethics, Section of Legal Medicine, Università Cattolica del Sacro Cuore, Largo Francesco Vito 1, Rome, Italy; 3 Fondazione Policlinico Universitario A. Gemelli IRCCS, Largo Agostino Gemelli 8, Rome, Italy; 4 Department of Translational Medicine, University of Ferrara, Via Fossato Di Mortara 70, Ferrara, Italy; 5 Department of Anti-Drug Policies, Collaborative Center for the Italian National Early Warning System, Presidency of the Council of Ministers, Rome, Italy; 6 REDs – Research and Expertise in antidoping Sciences, ISSUL – Institute of Sport Sciences, University of Lausanne, Lausanne, Switzerland; 7 Present Address: DoCoLab, University of Ghent, Block B, Ottergemsesteenweg 460, 9000 Ghent, Belgium

**Keywords:** Synthetic cannabinoids, Metabolite identification, APP-CHMINACA, PX-3, ICR-CD1 mice

## Abstract

**Purpose:**

The metabolic pathways of APP-CHMINACA were characterized to select the markers of intake for implementation into analytical assays used by the clinical and forensic communities. We have combined the evidences obtained by both in vitro experiments and administration studies on mice.

**Methods:**

APP-CHMINACA was incubated with either human or mouse liver microsomes. Urine and blood samples were collected at different time points from mice after injection of a 3 mg/kg dose of the test compound. Samples were analyzed using liquid chromatography-tandem mass spectrometry.

**Results:**

The in vitro studies allowed to isolate eight different metabolic reactions, formed by two metabolic routes, with no differences between human and mouse liver microsomes. The main biotransformation route involved the hydrolysis of the distal amide group and the subsequent hydroxylation on the cyclohexyl-methyl ring. The second route involved multiple hydroxylation of the parent compound, followed by reduction to generate minor metabolites. In blood samples, the most abundant substances identified were APP-CHMINACA unchanged and the metabolites formed by the hydrolysis of the distal amide together with its hydroxylated products. In urine samples, four metabolites formed following the hydroxylation of the distal amide hydrolysis metabolite were detected as the most abundant and long-term metabolites.

**Conclusions:**

The outcomes of our study showed that the most suitable markers to detect the intake of APP-CHMINACA in blood and urine samples in the framework of toxicological, clinical and forensic investigations were the metabolite formed by the hydrolysis of the distal amide and its hydroxylated products.

## Introduction

Synthetic cannabinoids (SCs) are psychoactive compounds pharmacologically related to the natural cannabinoids found in *Cannabis sativa* (i.e., Δ-9- tetrahydrocannabinol, Δ-8-tetrahydrocannabinol cannabinol, and cannabidiol [1, 2]). The family of SCs is a chemically heterogenous class of designer psychoactive substances and comprises compounds with remarkable differences in their molecular structures and in their biophysico-chemical properties. All SCs are, however, characterized by similar effects on the central nervous systems [3, 4], based on their interaction with the endogenous cannabinoid receptors CB_1_ and CB_2_. Although SCs were initially designed and synthesized as potential novel therapeutic agents, they rapidly emerged on clandestine market as a potent alternative to natural cannabis [5], with health risk related to their abuse [6].

The choice of many different products on the market, as well as the easy availability on the web and the potent effects on the central nervous system, have made SCs the most abused and chemically heterogenous class of novel psychoactive substance (NPS) [3, 7, 8]._._ Products belonging to the first-generation of SCs were sold with the name of Spice, a smokable herbal blend containing a mixture of different compounds, such as JWH-018, CP 47,497, JWH-073 and HU-210 [9]. In the following years, an increasing variety of SCs were synthesized and sold with variable effects on CNS, some of the most known ascribable to narcotic and hallucinogenic effects, and, to a lesser extent, stimulant effects [10, 11]

Prevalence studies indicated that the abuse of synthetic cannabinoids was an issue of relevance also in sport^12^, ^13^ : since 2010, the use of this class of NPS was prohibited, only if administered in competition, by the World Anti-doping Agency (WADA), included in the section S8 “Cannabinoids” of the list of prohibited substances and method updated at least annually by the WADA itself. [14].

Clinical, forensic, and anti-doping laboratories still face several challenges in the detection of the intake of SCs from the analysis of biological matrices. For indeed, the production of commercially available certified reference standards of the parent drug usually requires 6–24 months from the identification of a new SC [15]; furthermore, the extensive metabolism that characterizes the parent compound makes it no longer detectable in blood and/or urine already a few hours after the intake [16, 17]. In vitro and in vivo metabolism studies become, therefore, the most effective experimental strategy to collect information necessary to detect their abuse, not only to select the most appropriate marker(s) of intake, but also to assess the potential toxicological risks linked to their intake. This comprehensive approach plays a vital role in establishing an effective strategy to predict the metabolic processes of compounds yet to be studied. [18].

The present study focuses specifically on APP-CHMINACA (*N*-[(1S)-2-amino-2-oxo-1-(phenylmethyl)ethyl]-1-(cyclohexylmethyl)-1H-indazole-3-carboxamide), a third-generation SC also known as PX-3. APP-CHMINACA belongs to the subclass of indazole carboxamide-type SCs, one of the largest classes of SCs reported in clinical cases [19]. Its structure is characterized by a carboxamide linker group which links the indazole core to an APP moiety. The tail group is a cyclohexylmethyl (CHM) ring linked in the 1-position of the indazole core. APP-CHMINACA was synthesized for the first time by Buchler et al. for Pfizer in 2009 as potential therapeutic drugs where only the (S)-enantiomer was included in the patent. Indeed, APP-CHMINACA is a potent CB_1_ and CB_2_ receptor agonists (K_*i*_: 9.81 nM and 4.39 nM, respectively) [19]. And the activity of the (S)-enantiomer was proved to be much more effective than that of the (R)-enantiomer on CB_1_ (S/R EC_50_ = 0.0074) [20, 21].

The appearance of APP-CHMINACA in the market was suspected for the first time in 2015 after the Belgian Customs authorities seized a powder labeled as “white pigments”, initially delivered from China, which was found to contain APP-CHMINACA and another SC, methyl (2S)-2-{[1-(5-fluoropentyl)-1H-indazol-3-yl]formamido}-3-methylbutanoate, generally known as “5F-AMB” [22]. The illicit use of APP-CHMINACA was confirmed also by its detection in paraphernalia and product samples, together with other SCs, from patient affected by adverse effects compatible with those of SCs in Anchorage, Alaska (USA) [23]. The in vitro metabolic profile of APP-CHMINACA was defined for the first time by Cooman et al. [24] and later by Presely et al. [19], following incubation with human liver microsomes (HLM). The main metabolic reaction involved the hydrolysis of the distal amide followed by hydroxylation of the CHM moiety with five metabolites detected after the incubation. Although, the most abundant metabolite found in both studies was product of the hydrolysis of the distal amide, the authors suggested that this metabolite could undergo further extensive metabolism in vivo and therefore cannot be considered a suitable marker of intake.

The aim of this study was to elucidate the in vitro and in vivo phase I metabolic profile of APP-CHMINACA, to select the most suitable marker(s) of intake to be monitored in the analytical procedures of clinical and forensic laboratories. Since controlled excretion studies on human subjects would clearly be unacceptable, we have used both in vitro and animal models to trace the biotransformation reaction of APP-CHMINACA. More specifically, human and mouse liver microsomes were selected as for in vitro metabolism studies and ICR-CD1 mice were selected for the in vivo investigation. Urine and blood samples were collected up to 24 h and up to 300 min, respectively, from the administration of 3 mg/kg dose of APP-CHMINACA by injection. To the best of our knowledge this is the first study in which the metabolic profile of APP-CHMINACA was characterized in vivo and suitable markers of intake were selected in both blood and urine samples.

## Experimental

### Chemicals and reagents

APP-CHMINACA and AB-CHMINACA (*N*-[(2S)-1-amino-3-methyl-1-oxobutan-2-yl]-1-(cyclohexylmethyl)indazole-3-carboxamide, used as internal standard), were purchased from LGC standards (Settimo Milanese, Milano, Italy) and stored in methanolic solution (final concentration 1 mg/mL). Reagents and solvents (i.e., formic acid, sodium phosphate, sodium hydrogen phosphate, potassium carbonate, potassium hydrogen carbonate), all analytic grade, were purchased from Sigma-Aldrich (Milano, Italy). Ultra-purified water was by Milli-Q system (Millipore, Vimodrone, Milano, Italy). Human liver microsomes (HLM) and mice liver microsomes (MLM) and all the reagents used for the in vitro metabolism experiments (i.e., sodium phosphate buffer, NADPH regenerating system, NADP^+^, glucose-6-phosphate, and glucose-6-phosphate dehydrogenase) were supplied by Corning Incorporated (Milan, Italy).

### Liquid chromatography–mass spectrometry assays

The chromatographic separation was carried out using a Waters (Milford, MA, USA) Acquity I-Class UPLC® system equipped with a SUPELCO Discovery C18 column (2.1 mm × 150 mm 5 µm, Sigma-Aldrich, Milano, Italy). Solvents were: ultrapurified water (eluent A) and acetonitrile (eluent B), both containing 0.1% formic acid. The gradient program started at 15% B and increased linearly to 40% B over 7 min, and then after 3 min to 100% B over 1 min. The column was flushed for 3 min at 100% B and finally re-equilibrated at 15% B for 4 min. The flow rate was set to 250 µL/min.

The detection was performed by a triple quadrupole mass spectrometer (QTRAP 5500, Sciex, Milano, Italy) with an electrospray ionization (ESI) source. The mass spectrometric conditions were: ESI source operate in positive ion mode using a curtain gas pressure of 25 psi, an ion source temperature of 500 °C, an ion source gas 1 pressure of 45 psi, an ion source gas 2 pressure of 55 psi, a declustering voltage of 60 V, an entrance potential of 10 V and a needle voltage of 5500 V. Multiple reaction monitoring (MRM) was used as the acquisition mode (see Table 1 for the precursor ions, product ions and collision energies selected for each metabolic reaction). For the MRM collision-induced dissociation (CID), nitrogen was used as the collision gas at 5.8 mPa, obtained from a dedicated Parker-Balston nitrogen generator system (model 75-A74) with 99.5% gas purity (CPS Analitica Milano, Italy). All aspects of instrument control, method setup parameters, sample injection, and sequence operation were controlled by Analyst software version 1.5.1. (Sciex, Milano, Italy).

**Table 1:** Precursor ions, product ions (ions selected for MRM quantitation are underlined), and collision energies included in the MRM acquisition method used for the detection of APP-CHMINACA and its metabolic reactions.

Metabolic reaction	Precursor ion (*m/z*)	Product ions (*m/z*)*	Collision energy (eV)
APP-CHMINACA	405	119,145,241,360	50,50,30,30
Distal amide hydrolysis (M1)	406	119,145,241	50,50,30
Distal amide hydrolysis and ketone (CHM) formation (M2)	420	145,255,374	50,30,30
Mono-hydroxylation (CHM) (M3)	421	119,145,257,376	50,50,30,30
Distal amide hydrolysis + hydroxylation (CHM) (M4)	422	119,145, 239, 257	50,50,30,30
Hydroxylation and ketone (CHM) formation (M5)	435	119,145,253,271	50,30,30,30
Di-hydroxylation (CHM) (M6)	437	119,145,273,392	50,50,30,30
Di-hydroxylation and ketone (CHM) formation (M7)	451	119,145,271	50,50,30
Tri-hydroxylation (CHM) (M8)	453	119,145,271,289	50,30,30,30

^*^the product ions underlined were used to define the profile in the biological matrices selected

### Drug preparation and dose selection

APP-CHMINACA was initially dissolved in absolute ethanol (2%) and Tween 80 (2%) and brought to the final volume with saline (0.9% NaCl). The solution made with ethanol, Tween 80 and saline was also used as the vehicle. Drug was administered by an intraperitoneal route at a volume of 4 ul/g. A dose of 3 mg/kg of APP-CHMINACA was chosen based on our previous study on SCs with indazole core [25, 26]

### Protocol for the in vitro metabolism studies

APP-CHMINACA was incubated in the presence of either HLM or MLM to characterize the phase I metabolic profile and to compare it with the evidence obtained by the administration studies on mice. The in vitro protocol was based on similar protocols already in use in our laboratory to perform metabolism studies [27–33]. The substrate concentration and incubation time were optimized with the aim to produce the maximum yield of the metabolites.

Incubation studies were carried out at 37 °C and samples were collected at different time points (15, 30, 60, 120 min). At the end of the incubation period, 250 μL of acetonitrile were added to stop the phase I reactions. Each set of assays also included a negative control sample without HLM or MLM, to assess the potential non-enzymatic reactions, and a negative control sample constituted by all the components of the reaction mixture, but without APP-CHMINACA**.** All incubations were performed in phosphate buffer (0.1 M, pH 7.4). The final incubation medium contains: 10 µM of substrate, 0.5 mg/mL of proteins, 3.3 mM of magnesium chloride, 1.3 mM of NADP^+^, 3.3 mM of glucose-6-phosphate, and finally 0.4 U/mL of glucose-6-phosphate dehydrogenase. Each incubation experiment was processed in triplicate.

### Protocols for the in vivo experiments

#### Animals

ICR (CD-1^®^) mice weighing 30–35 g (Centralized Preclinical Research Laboratory, University of Ferrara, Italy) were group housed (4/cage; floor area: 80 cm^2^/mouse; minimum enclosure height: 12 cm), exposed to a 12:12-h light–dark cycle (light on at 6:30 AM) at a temperature of 20–22 °C and humidity of 45–55% and provided ad libitum access to food (Diet 4RF25 GLP; Mucedola, Settimo Milanese, Milan, Italy) and water.

#### Urine samples collection

Urine specimens were collected from mice individually placed inside metabolic cages (Ugo Basile SRL, Gemonio [VA], Italy) with free access to water and food. The urinary excretion profile was studied through protocols already described in previous publications [28, 30, 31, 33]. A group of five mice were administered with a single dose of 3 mg/kg of APP-CHMINACA and urine samples were collected starting at 9:00 AM for each mouse. The samples were collected in pool every 2 h in the first 6 h and in two vials for 6–12 h and 12–24 h range, respectively. Urine blank samples from the mice control group were also collected in the same time intervals.

#### Blood samples collection

Blood collection was carried out at 30, 180, 240 and 300 min. Blood samples were collected by submandibular blood collection technique, into 1 mL vials containing EDTA (4 mg/mL of blood). After each blood withdrawal, an equal volume of saline solution was subcutaneously injected in mice to maintain volume and osmotic homeostasis. Urine samples were stored at -20 °C until the analysis. The blood samples were centrifuged at 2700 × *g* for 15 min; the supernatant was then stored at -20 °C until the analysis.

#### Sample pre-treatment

For the urinary excretion studies, a volume of 50 μL of urine samples was added with 50 μL of internal standard solution (ISTD, AB-CHMINACA standard solution final concentration 100 ng/mL) and 20 μL of β-glucuronidase. The samples were then buffered with 200 μL of phosphate buffer (0.8 M, pH 7.4), incubated for 60 min at 50 °C and finally extracted with 5 mL of ethyl-acetate.

For blood metabolic profile, 50 μL of sample were added with 200 μL of water and 200 μL of acetonitrile and centrifugated at 37,000 × *g* for 10 min after mixing for 5 min. The supernatant was then collected and added with 50 μL of internal standard solution (final concentration 100 ng/mL) and 100 μL of phosphate buffer (0.8 M, pH 7.4). Samples were then extracted with 5 mL of ethyl-acetate.

For both protocols, the organic solvent was then dried under nitrogen flow at 40 °C and the dry residue was redissolved in 50 μL of mobile phase and an aliquot of 10 µL was injected into the LC–MS/MS systems.

#### Profile of the parent compound and its metabolites in the biological matrices selected

The estimation of the levels of APP-CHMINACA was carried out by generating calibration curves using a 1/x weighted least-squared regression. The calibration curves were prepared using negative samples from mice. The sample volume and the preparation protocol used are those described for the excretion study samples. The negative samples were spiked at different concentrations of APP-CHMINACA standard solution, using AB-CHMINACA as internal standard. All the levels of the calibration curve were prepared in duplicate. The concentration ranges were chosen based on the analysis of mice blood samples. Two different calibration curves were used, the first in the range 0.75–6.0 ng/mL, the second in the range 6–100 ng/mL. The limit of detection (LOD) and quantification (LOQ) were estimated by linear regression analysis. Due to the lack of certified reference standards for the metabolic products identified, the ratio between the peak area of the compound detected and the internal standard was used to estimate the relative abundance of each metabolite.

## Results

### Mass spectrometric data

**Fig. 1:**
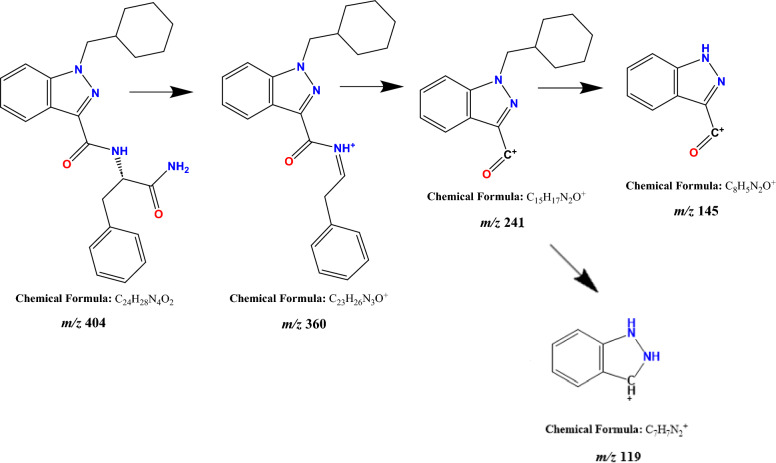
Characteristic fragmentation pathway of APP-CHMINACA and proposed fragmentation pattern

The characteristic fragmentation pattern of APP-CHMINACA was defined by the injection of a standard solution of the drug at a concentration of 10 µg/mL dissolved in mobile phase. The protonated molecule for APP-CHMINACA was found at *m/z* 405. The fragmentation pattern was defined at different collision energies (i.e., CE 20, 30, 40, 45, 50 and 60 eV) with the triple quadrupole analyzer (QqQ) operating in product ion scan mode. At low collision energies, the loss of the distal amide group was detected as the fragment ion at *m/z* 360 (CE 30 eV), followed by subsequent fragmentation to form indazole-acylium cation linked to CHM moiety at *m/z* 241 (CE 30 eV). At higher collision energies, indazole acylium cation was detected at *m/z* 145 (CE 50 eV), a characteristic fragment ion of the indazole-3-carboxamide cannabinoid. At the same CE, the indazole core alone was detected at *m/z* 119. The structures of the detected fragments ions are reported in Fig. 1.

The characteristic ion transitions used to set up the MRM acquisition method for the metabolic products were obtained by selecting the characteristic fragmentation routes (structural markers) found in the product ion spectrum of APP-CHMINACA, assuming a similar fragmentation behavior also for the hypothesized phase I metabolites. Metabolic reactions were hypothesized based on known information for APP-CHMINACA [19, 24] and similar compounds [34–38] (see Table 1 for characteristic MRM ion transitions and collision energies selected).

### Characterization of APP-CHMINACA metabolic profile and selection of the markers of intake

In vitro and in vivo samples were analyzed by using the analytical procedure described in the experimental part. The characterization of the metabolic profile was based on the presence or absence of the characteristic product ions included in the multiple reaction monitoring acquisition method developed in this study.

#### In vitro investigation

**Fig. 2:**
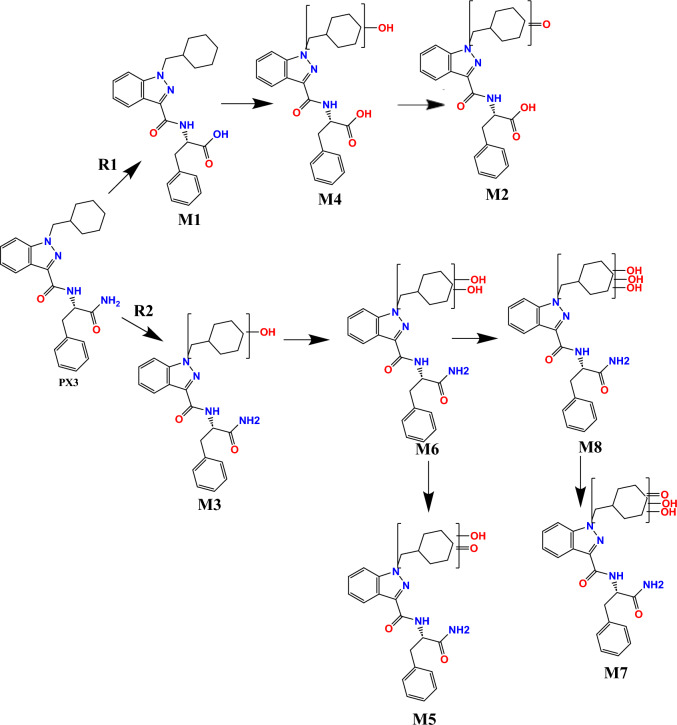
Metabolic reactions identified for APP-CHMINACA by two different metabolic routes (R1 and R2). R1: Distal amide hydrolysis (M1), distal amide hydrolysis and ketone formation on CHM (M2), distal amide hydrolysis and mono-hydroxylation on CHM (M4). R2: mono-hydroxylation on CHM (M3), mono-hydroxylation and ketone formation on CHM (M5), di-hydroxylation on CHM (M6), di-hydroxylation and ketone formation on CHM (M7), tri-hydroxylation on CHM (M8)

The APP-CHMINACA standard solution was incubated with either HLM or MLM. After 1 h of incubation of 10 μM of the target analyte at 37 °C, eight different metabolic reactions were detected: hydrolysis of the distal amide (M1), hydrolysis of the distal amide followed by the ketone formation on CHM (M2), mono-hydroxylation on CHM (M3), hydrolysis of the distal amide and mono-hydroxylation on CHM (M4), mono-hydroxylation and ketone formation on CHM (M5), di-hydroxylation on CHM (M6), di-hydroxylation and ketone formation on CHM (M7), tri-hydroxylation on CHM (M8) (see Fig. 2 for the hypothesized structures and Table 1 for the metabolic reactions identified and their respective precursor ions, product ions and collision energies).

Two different metabolic pathways were identified, confirming the results reported by previous investigators [19, 24]. The first pathway involved mainly the hydrolysis of the distal amide to carboxylic acid (M1) followed by hydroxylation in different positions of the CHM portion (M4.1-M4.5). Dehydrogenation of M4 metabolites to ketone (M2) was identified as a minor metabolic reaction of this route (see again Fig. 2). The second metabolic pathway, instead, involved multiple hydroxylation and dehydrogenation of the parent drug leading to the formation of the following metabolic products: mono-hydroxylation (M3), di-hydroxylation (M6), di-hydroxylation followed by mono-dehydrogenation (M5), tri-hydroxylation (M8), and finally tri-hydroxylation followed by mono-dehydrogenation (M7) (see again Fig. 2). A strong interspecies correlation was observed between HLM and MLM, suggesting that the phase I metabolic reactions found in mice could also be representative of the phase I biotransformation pathways of APP-CHMINACA in humans.

**Fig. 3:**
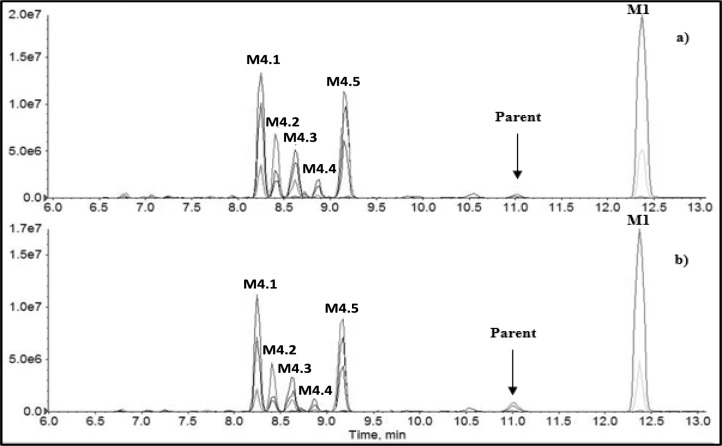
Extracted chromatogram of APP-CHMINACA and the principal metabolites identified after 1 h by the incubation with MLM **a** and HLM **b**. APP-CHMINACA distal amide hydrolysis (M1), distal amide hydrolysis and mono-oxidation (CHM) (M4.1–5)

The extracted chromatograms of representative samples obtained after incubation of the target compound in the presence of either HLM or MLM are reported in Fig. 3.

#### In vivo studies

##### Blood

**Fig. 4:**
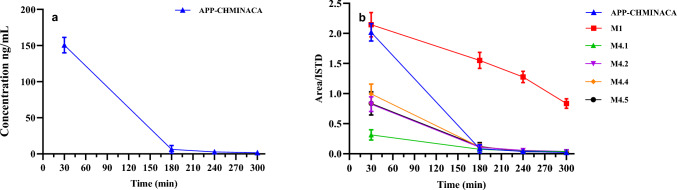
Levels of APP-CHMINACA in blood samples over the range of 30–300 min by the intraperitoneal administration of a 3 mg/kg dose (**A**)**.** Blood profile of APP-CHMINACA and its main metabolites over the range of 30–300 min by the intraperitoneal administration of a 3 mg/kg dose of APP-CHMINACA (**B**)

Most of the metabolic reactions identified in the vitro experiments were detected in blood samples, together with the parent compound (see Table 2). M1, M4.1, M4.2, M4.4 and M4.5 metabolites were the most abundant and were detectable throughout the entire range explored (30–300 min), being M1 the main metabolite detected together with the parent compound (see Figs. 4). M4.3, M3.1, M3.2, M3.3 and M3.4 were not detectable throughout the entire range explored (30–300 min). The products of the other metabolic reactions (that are, M2, M5, M6, M7 and M8) were, instead, detected in traces (see Table 2). The blood levels of APP-CHMINACA are reported in Fig. 4a, while the profiles of APP-CHIMINACA and its main metabolic products are reported in Fig. 4b. As it can be seen the parent compound, and its metabolites, showed a maximum in the first collection (30 min). A pronounced decrease of the levels of the parent drug, M4.1, M4.2, M4.4 and M4.5 was observed at 180 min after drug administration, whereas the levels of M1 showed a less marked decrease throughout the entire range explored (30–300 min). The above observations suggest that M1 can be considered the most suitable diagnostic marker to detect the intake of APP-CHMINACA in blood samples.

**Table 2:** Metabolites detected after incubation with HLM and MLM as well as in blood and urine samples of mice. Each metabolite identified was reported with the respective biotransformation and retention time (RT)

Biotransformation	Metabolite	RT (min)	HLM	MLM	blood	Urine
//	Parent	11.02	±	±	+ + +	−
Distal amide hydrolysis	M1	12.38	+ + +	+ + +	+ + +	−
Distal amide hydrolysis and ketone (CHM) formation	M2.1	8.749	±	±	±	±
	M2.2	8.994	±	±	±	±
Mono-hydroxylation (CHM)	M3.1	7.718	±	±	+	±
	M3.2	7.903	−	−	+	±
	M3.3	8.141	−	−	+	±
	M3.4	8.617	±	±	+	−
Distal amide hydrolysis and hydroxylation (CHM)	M4.1	8.247	+ + +	+ + +	+	+ + +
	M4.2	8.432	+ +	+ +	+ +	+ + +
	M4.3	8.617	+ +	+ +	−	−
	M4.4	8.881	+	+	+ +	+ + +
	M4.5	9.172	+ + +	+ + +	+ +	+ +
Hydroxylation and ketone (CHM) formation	M5	7.929	±	±	±	±
Di-hydroxylation (CHM)	M6.1	7.084	±	±	±	±
	M6.2	7.242	±	±	±	±
Di-hydroxylation and ketone (CHM) formation	M7.1	6.877	±	±	±	±
	M7.2	6.991	±	±	±	±
Tri-hydroxylation (CHM)	M8.1	6.264	±	±	±	±
	M8.2	6.504	±	±	±	±

+  +  + most abundant metabolite

+  + abundant metabolite

+ minor metabolite

± metabolite present in traces

− metabolite undetected

##### Urine

The analyses carried out in urine lead to the identification of a metabolic profile very similar to those observed in blood and by the in vitro studies. All the metabolic reactions identified in the other matrices were also identified in urine except for the M1 and M3.4. The metabolites produced by the hydroxylation of M1 are the main metabolites observed: M4.1, M4.2, M4.4 and M4.5, also for urine sample, M4.3 was not detectable throughout the entire range explored. The other metabolic reactions (M2, M5, M6, M7 and M8) were detected in traces (see Table 2).

**Fig. 5:**
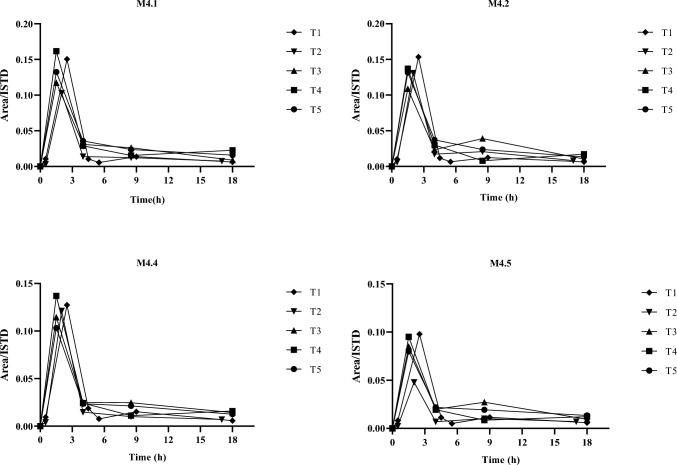
Excretion profile of the main metabolites found in urine after the intraperitoneal administration of a 3 mg/kg dose of APP-CHMINACA

Figure 5 reports the excretion profiles of the most abundant metabolites found in urine samples (M4.1, M4.2, M4.4 and M4.5). As it can be seen all the four metabolites reached the maximum excretion after 1.5–2.5 h from drug administration and are detectable throughout the range explored and therefore appear to be suitable diagnostic markers to detect the intake of APP-CHMINACA in urine samples in the framework of toxicological, clinical and forensic investigations.

## Discussion

We have studied the phase I metabolic pathways of APP-CHMINACA by integrating the experimental evidences obtained by in vitro studies and by controlled administration studies on mice. For indeed, whenever controlled excretion studies on human volunteers are ethically unacceptable, a combination of in vitro and animal studies may supply a significant amount of information to obtain a reliable prospect of the actual phase I metabolic processes [39]. Our aim was to select and propose the most appropriate marker(s) of intake, allowing to trace the consumption of this NPS from the analysis of wither urine and/or blood samples. We have first performed in vitro studies on HLM and MLM, to assess the interspecies correlation. In vivo studies on mice were then performed to select the target analytes to be considered in the screening assays used by the clinical and forensic communities to monitor the intake of NPS. Indeed, blood is the biological fluid of choice to monitor the levels of an exogenous compound after a recent intake, also allowing to know whether the subject is still under its pharmaco-toxicological effects, whereas urine is a more appropriate biological fluid whenever a more prolonged window of detectability is required.

The in vitro protocols and the metabolite characterization procedures used in the current study have been already successfully applied in the past to elucidate the in vitro phase I metabolic profile of several classes of prohibited substances [27–33, 40–43]. In some cases, the in vitro investigations were accompanied by in vivo studies via LC–MS analysis of biological specimens (i.e. blood and urine) [30, 31, 40, 41, 43]; high correlations between the metabolites identified in vitro and those detected in vivo were reported proving the broad applicability of such a combined experimental strategy.

In this study, eight different phase I metabolic reactions, formed by two metabolic routes, were characterized, with no differences between human and mouse liver microsomes. The main biotransformation route involved the hydrolysis of the distal amide group and the subsequent hydroxylation on the cyclohexyl-methyl ring. The second route involved multiple hydroxylation of the parent compound, followed by reduction to generate minor metabolites. Our findings are consistent with those reported by Cooman et al. [24] and Presely et al. [19], following incubation with HLM. In both these studies, the metabolic products proposed as markers of intake were the hydroxylated metabolite, the distal amide hydrolysis product, and its hydroxylated metabolite. The evidences of our in vivo partially confirm those findings. Indeed, the most abundant substances identified in blood samples, were APP-CHMINACA unchanged and the metabolites formed by the hydrolysis of the distal amide, together with its hydroxylated products, whereas in urine samples, the most abundant and long-term metabolites were those metabolites formed following the hydroxylation of the distal amide hydrolysis metabolite. Based on these observations, our proposal is to consider the M4 metabolites generated from the hydroxylation of the distal amide hydrolysis metabolite as the most appropriate markers of use in urine samples and the distal amide hydrolysis metabolite (M1) as the main marker of intake in blood samples.

Our metabolic study was limited to the phase I biotransformation reactions, without considering phase II metabolism. However, being the class of SCs reported to be mostly excreted as glucurono-conjugates, our target compounds would still be adequate, provided that the hydrolysis with β-glucuronidase is included in the sample pretreatment process of the screening procedures followed by clinical and forensic laboratories.

## Conclusion

To the best of our knowledge, this is the first study focused on the metabolic profile of APP-CHMINACA, combining the parallel, experimental observations obtained by both in vitro and in vivo metabolism studies. The results obtained in blood and urine collected from mice after the administration of a dose of 3 mg/kg showed that APP-CHMINACA metabolizes via two different metabolic pathways, the main one involving hydrolysis of the amide group and subsequent hydroxylation of the CHM moiety, confirming the results reported by previous investigators after in vitro studies. We recommend as most appropriate markers of intake, the M4 metabolites generated from the hydroxylation of the distal amide hydrolysis metabolite in urine samples and the distal amide hydrolysis metabolite (M1) in blood samples. The results of this study can be used as a guide to predict the metabolic pathways of similar compounds and to synthesize the reference standards of the selected markers of intake.
